# Gluten-Free Snacks with Micronized and Freeze-Dried Red Potatoes: Nutritional and Pro-Health Values

**DOI:** 10.3390/molecules30091957

**Published:** 2025-04-28

**Authors:** Dorota Gumul, Marek Kruczek

**Affiliations:** Department of Carbohydrate Technology and Cereal Processing, Faculty of Food Technology, University of Agriculture in Krakow, 122 Balicka St., 30-149 Krakow, Poland; marek.kruczek@urk.edu.pl

**Keywords:** micronized red potatoes, gluten-free extrudates, antioxidant activity

## Abstract

The application of micronization to previously freeze-dried red potatoes significantly increased their polyphenol content and antioxidant potential. As a result, they became a valuable additive for enriching gluten-free snacks with bioactive compounds. The aim of this study was to assess the health-promoting potential as well as the content of polyphenols, phytosterols, and vitamin E in gluten-free extrudates, also referred to as gluten-free snacks, with the addition of 10% to 40% freeze-dried and micronized red potatoes. Additionally, the study examined color parameters and nutritional composition, including dietary fiber content. It was found that the extrudates obtained from micronized and freeze-dried red potatoes were characterized by high nutritional value but, most importantly, a strong health-promoting potential due to their exceptionally high content of phenolic acids and anthocyanins, which contributed to their remarkable antioxidant activity. Snacks enriched with freeze-dried and micronized red potatoes contain significantly higher levels of protein (3- to 14-fold increase), ash (4.5- to 22.5-fold increase), and soluble dietary fiber fraction (10- to 26-fold increase) compared to the control sample. Moreover, these snacks exhibited very high concentrations of chlorogenic, cryptochlorogenic, and neochlorogenic acids, as well as elevated levels of pelargonidin and peonidin glycosides—polyphenolic compounds that were not detected in the control sample. These snacks contained substantial amounts of tocopherols and phytosterols, such as stigmasterol and beta-sitosterol (3- to 10-fold increase compared to the control). The study conclusively demonstrated that the 40% addition of freeze-dried and micronized red potatoes to gluten-free extrudates ensures the development of an innovative product with excellent health benefits and strong antioxidant activity.

## 1. Introduction

Red and purple potatoes are the third-largest source of polyphenols in the human diet, following apples and citrus fruits [1]. This explains the growing interest from scientists, producers, and consumers in these potatoes, due to the bioactive compounds they contain, particularly polyphenols. Many authors [2,3,4,5] consider potatoes a functional food due to their polyphenol content. Among the polyphenols in potatoes, phenolic acids are the most abundant group found in potato tubers [6,7,8]. The dominant phenolic acid is chlorogenic acid, followed by its derivatives, neo- and cryptochlorogenic acids [8,9,10]. According to Rytel et al. [7], potatoes with colored flesh are richest in chlorogenic and neochlorogenic acids, with other acids present in smaller amounts. Similarly, Deusser et al. [11] observed that chlorogenic acid and its two isomers dominate in both light and colored-flesh potatoes, with other acids, such as caffeic acid, mostly found in the skin. Akyol et al. [6] and Mäder et al. [12] also noted that potatoes with different flesh colors are low in phenolic acids like caffeic, coumaric, ferulic, sinapic, and gallic acids.

Another important group of polyphenolic compounds in potatoes are flavonoids, including catechin, epicatechin, kaempferol, and rutin [10]. Lewis et al. [13] found that red- or purple-flesh potatoes have twice the flavonoid concentration compared to light-flesh potatoes, with higher amounts found in the skin (around 900 mg/100 g in purple potatoes and 500 mg/100 g in red potatoes). Anthocyanins, a subgroup of flavonoids, are present only in red and purple potatoes and/or their skin, with concentrations ranging from 5.5 to 35 mg/100 g [14]. These anthocyanins are acylated with phenolic acids, mainly ferulic and p-coumaric acids. Purple potatoes contain petunidin and malvidin 3-rutinoside-5-glucoside, acylated with p-coumaric and ferulic acids, while red potatoes are rich in pelargonidin and peonidin 3-rutinoside-5-glucoside, also acylated with p-coumaric and ferulic acids [15]. These compounds are more stable during thermal processing in food production compared to anthocyanins in colored fruits.

The polyphenols in plants exhibit a wide range of biological activities, including antibacterial, antiviral, antioxidant, diuretic, and detoxifying effects. Flavonoids inhibit platelet aggregation, reduce arterial muscle tension, improve endothelial function, and prevent cancer by limiting DNA damage and tumor growth. They also show anti-atherosclerotic effects, benefiting the cardiovascular system and reducing the risk of coronary artery disease [16,17]. The anticancer effects of anthocyanins (mainly delphinidin, pelargonidin, petunidin, and malvidin) target hormone-dependent cancers, such as breast cancer in women [18] and lymph node cancers [19]. Chlorogenic acid, dominant in potatoes, helps prevent degenerative diseases, coronary heart disease, and exhibits anticancer, antiviral, and antibacterial properties, while also lowering blood pressure [20,21]. Chlorogenic acid is a strong, selective inhibitor of matrix metalloproteinase (MMP), an angiogenic enzyme involved in tumor invasion and metastasis [22]. It also slows glucose release into the bloodstream [23], potentially lowering the glycemic index (GI) of potatoes. Therefore, potatoes with a lower GI are beneficial for diabetic patients and may reduce the risk of type II diabetes [24].

Apart from polyphenols, red and purple potatoes also contain other bioactive compounds with potential health benefits, such as phytosterols, which are natural components of plant cell membranes [25,26]. Phytosterols have been shown to play a significant role in the prevention of cardiovascular diseases by lowering levels of low-density lipoprotein (LDL) and very-low-density lipoprotein (VLDL) cholesterol fractions [27,28,29]. Moreover, phytosterols have also been reported to act as effective preventive agents against gastrointestinal (GIT) dysfunctions, including inappropriate motor activity, gastric ulcers, and inflammatory bowel disease (IBD), as well as liver and pancreatic disorders. The above-mentioned diseases are the result of poor diet, pharmacological treatments, and pathogenic infections [29].

To effectively utilize the health potential of red and purple potatoes, they must be appropriately incorporated into food technology processes. Freeze-drying red-fleshed and red-skinned potatoes, followed by their micronization and subsequent use in the extrusion process, appears to be a valuable approach. This could result in the creation of snacks with a high antioxidant potential. Micronization, commonly used in the pharmaceutical industry, has shown significant potential in the food industry in recent decades. Studies indicate that micronization, which reduces particle size, positively affects the functionality and physicochemical properties of raw materials and enhances the bioavailability of polyphenols [30,31,32]. Extrusion, on the other hand, is an industrial technique that creates new products, ensuring they are free from microbiological contamination, have low acrylamide content, and, most importantly, an extended shelf life [33]. Thus, combining micronization and extrusion of red potatoes could produce snacks with significant health potential, as these two operations may increase the bioavailability of health-promoting compounds in red potatoes. Therefore, the aim of this study was to obtain extrudates (snacks) with freeze-dried red potatoes after micronization. The study also examined the impact of varying levels of freeze-dried and micronized red potato inclusion on the pro-health potential of the resulting snacks, including polyphenol content, quantitative and qualitative polyphenol profile, antioxidant activities, tocopherol, and phytosterol content. Additionally, the nutritional composition and color of these snacks were analyzed.

## 2. Results and Discussion

### 2.1. Characteristics of Freeze-Dried and Micronized Red Potatoes

The total polyphenol content (TPC) in freeze-dried and micronized red potatoes of the Magenta Love (ML) variety, determined using the Folin–Ciocalteu (F-C) reagent, was approximately 19.24 mg catechin/g of dry matter (DM).

**Figure 1:**
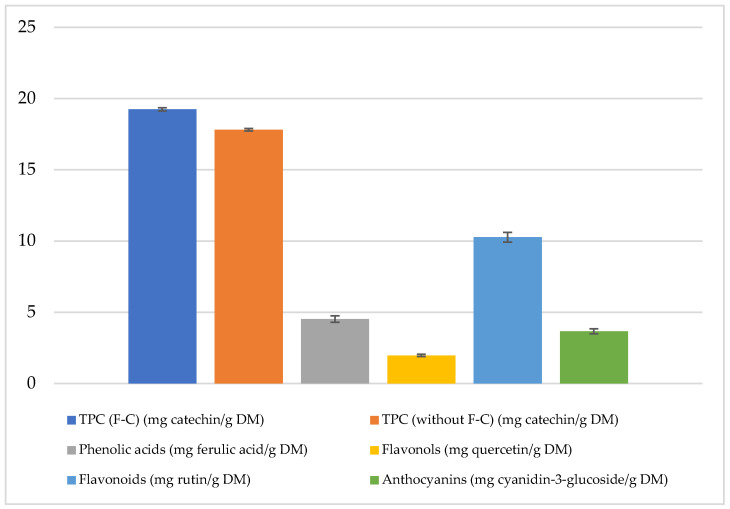
Phenolic compounds in freeze-dried and micronized red potatoes, variety ML (data presented as equivalents in mg/g DM for each compound).

The polyphenol content was 8% lower in the absence of the F-C reagent compared to the method using it. Phenolic acid content reached 4.53 mg ferulic acid/g DM, flavonoids 10.27 mg rutin/g DM, flavonols 1.98 mg quercetin/g DM, and anthocyanins 3.67 mg cyanidin-3-glucoside/g DM in freeze-dried and micronized red potatoes (Figure 1).

**Figure 2:**
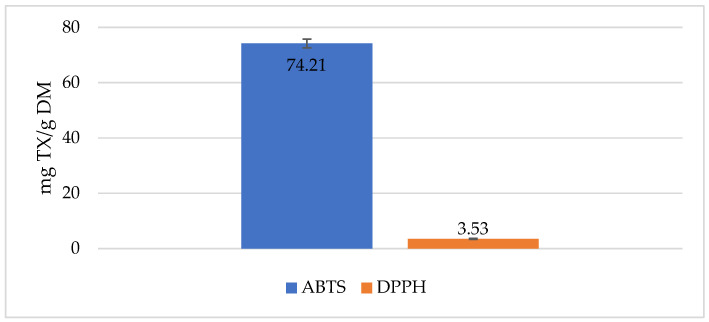
Antiradical activities of freeze-dried and micronized red potatoes, variety ML.

Antiradical activity against DPPH and ABTS radicals was 3.53 and 74.21 mg Trolox/g DM, respectively, for freeze-dried and micronized red potatoes (Figure 2).

An analysis of the phenolic profile (Table 1) revealed that the primary antioxidants among polyphenols in colored-flesh potatoes are phenolic acids, particularly chlorogenic acid and its isomers (cryptochlorogenic and neochlorogenic acid). These compounds constitute 86% of all phenolic compounds in potatoes (Table 1), aligning with previous studies reporting their contribution at approximately 90% in potato tubers [6,7,8]. Chlorogenic acid was the most abundant (892.02 mg/100 g DM), followed by neochlorogenic acid (287 mg/100 g DM) and cryptochlorogenic acid (195.07 mg/100 g DM). Thus, the amounts of neochlorogenic and cryptochlorogenic acids were approximately 3 and 4.5 times lower than chlorogenic acid in freeze-dried Magenta Love potatoes after their micronization. The lowest content among phenolic acids was found for p-coumaric acid glucoside (Table 1). Phenolic acids represent the largest group of phenolic compounds in potato tubers [6,7,8], with chlorogenic acid as the dominant compound, followed by its derivatives neo- and cryptochlorogenic acids [6,8,9,10,34]. According to Rytel et al. [7], potatoes with colored flesh are richest in chlorogenic and neochlorogenic acids, while other acids occur in smaller quantities. Similarly, Deusser et al. [11] noted that chlorogenic acid and its two isomers are dominant in both light- and colored-flesh potatoes, whereas other acids, such as caffeic acid, are more concentrated in the potato peel. Akyol et al. [6] and Mäder et al. [12] further confirmed that potato tubers, regardless of flesh color, have low levels of phenolic acids such as caffeic, coumaric, ferulic, synapinic, and gallic acids.

The results of this study support previous findings regarding the dominant presence of chlorogenic acid in red-fleshed potatoes (Table 1). The significant levels of chlorogenic acid in colored-flesh potato tubers are crucial due to its chemopreventive role. This phenolic acid has been shown to have protective effects against degenerative diseases, coronary diseases, and cancer, as well as antiviral, antibacterial, and blood pressure-lowering properties [20,21].

Anthocyanins are a highly valuable group of compounds due to their health-promoting effects. They exhibit anti-inflammatory, antiviral, and antibacterial properties [16]. Additionally, anthocyanins reduce the risk of cancer, coronary diseases, Alzheimer’s disease, and diabetes. They also improve night vision and reduce the risk of cataracts [17]. These compounds are abundant in red- and purple-fleshed potatoes, contributing to their high antioxidant and health-promoting potential. Anthocyanins form a specific subgroup within flavonoids. In red-fleshed potatoes, pelargonidin derivatives are the dominant anthocyanins [15,35,36]. Rodriguez-Saona et al. [37] reported a high content of pelargonidin-3-(caffeoyl) rutinoside-5-glucoside in these potatoes, noting its acetylation with p-coumaric and ferulic acids. Similarly, Lachman et al. [15] showed that red potatoes are rich in pelargonidin and peonidin, both acylated with p-coumaric and ferulic acids. In the Magenta Love variety of red potatoes, after freeze-drying and micronization, several pelargonidin derivatives were identified. These include pelargonidin-3-rutinoside-5-glucoside (9.74 mg/100 g DM) and pelargonidin-3-(caffeoyl)rutinoside-5-glucoside (10.34 mg/100 g DM). A significant amount of pelargonidin-3-(p-coumaroyl)rutinoside-5-glucoside (187.77 mg/100 g DM) and a small amount of peonidin-3-rutinoside-5-glucoside (0.49 mg/100 g DM) were also detected (Table 1). In comparison, Nemś et al. [36] reported pelargonidin-3-rutinoside-5-glucoside at 1.76 mg/100 g DM and pelargonidin-3-(p-coumaroyl)rutinoside-5-glucoside at 86.7 mg/100 g DM in the Herbie26 red-fleshed potato variety. Kita et al. [35] observed pelargonidin-3-rutinoside-5-glucoside levels ranging from 2.17 to 11.84 mg/100 g DM and pelargonidin-3-(caffeoyl)rutinoside-5-glucoside between 0.51 and 2.31 mg/100 g DM in similar plant material. The differences in anthocyanin content between the above-mentioned authors and the results presented in this study can be attributed to various factors such as climatic, soil, and agronomic conditions and most importantly, the application of the micronization process [15,25,35,36,38]. In this case, micronization significantly enhanced the extraction of polyphenols [31]. A similar increase in the content of polyphenols, flavonoids, and monomeric anthocyanins in tart cherry puree—by 61%, 46%, and 49%, respectively—was observed by Lukman et al. [39]. Lukman et al. [39] concluded that the increase in these bioactive compounds was caused by the micronization of tart cherry puree. Similarly, Różyło et al. [32] reported a 30–80% increase in polyphenol content following micronization of plant materials. Comparing the content of polyphenols, flavonoids, and anthocyanins in freeze-dried and micronized red potatoes of the Magenta Love variety analyzed in this study with our previous research [40] concerning freeze-dried but non-micronized Magenta Love red potatoes, it can be concluded that the application of micronization resulted in an increase in polyphenols, flavonoids, and anthocyanins by 17%, 18%, and 39%, respectively. This increase can be attributed to enhanced extraction of polyphenolic compounds following the micronization process. The use of a ball mill for micronization causes disintegration of plant cell walls, thereby releasing polyphenols and improving their extractability. These observations are consistent with previous studies demonstrating the positive effect of micronization on the improved extraction efficiency of polyphenols from plant materials [30,31,32,39].

Additionally, red potatoes contain 0.71 mg/100 g DM of alpha-tocopherol, 1.13 mg/100 g DM of stigmasterol, and 1.82 mg/100 g DM of sitosterol (Table 1).

**Figure 3:**
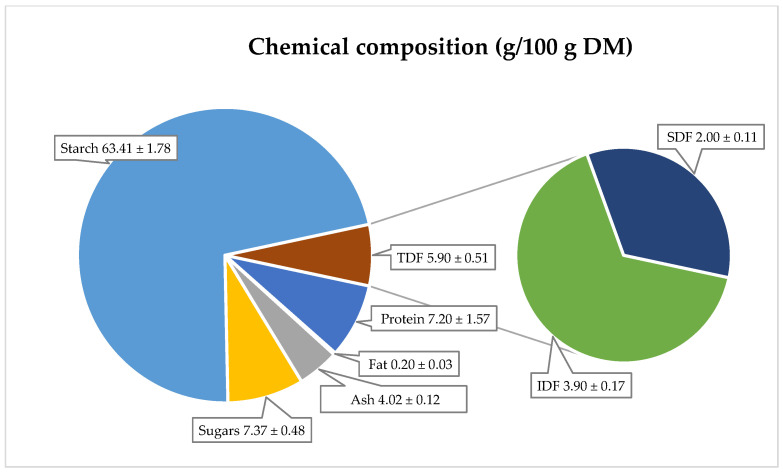
Chemical composition of freeze-dried and micronized red potatoes, variety ML Note: TDF—total dietary fiber, IDF—insoluble dietary fiber, SDF—soluble dietary fiber.

Potatoes are also recognized as a source of carbohydrates, high-quality protein, vitamins, and minerals [41,42]. Their nutritional composition is as follows: protein content—7.2 g, ash—4.0 g, fat—0.2 g, sugar—7.4 g, and starch—63.4 g per 100 g dry matter (Figure 3). The starch content in red-fleshed potatoes ranged from 15.8 to 17.9 g/100 g of fresh weight (equivalent to 63.2–71.6 g/100 g DM) [43]. The total sugar content, according to Kita et al. [35], was between 0.14 and 0.51 g/100 g fresh weight (equivalent to 0.56–2.04 g/100 g DM). These values are consistent with other literature data for red-fleshed potatoes.

Dietary fiber in potatoes is a chemically heterogeneous complex, consisting of insoluble and soluble fractions, each with distinct physiological effects. Insoluble fiber is recommended for preventing and treating colon disorders, such as chronic constipation, irritable bowel syndrome, hemorrhoids, and diverticulosis. Soluble fiber, on the other hand, exhibits hypocholesterolemic, hypoglycemic, and anticancer properties. The primary dietary sources of fiber include cereals, vegetables, and fruits [44,45,46]. In red potatoes, the total dietary fiber content is 5.9 g/100 g DM, with the soluble fraction at 2.0 g/100 g DM and the insoluble fraction at 3.9 g/100 g DM. However, there is limited literature on the fat, ash, protein, and fiber content in red-fleshed potatoes.

Additionally freeze-dried and micronized red potatoes were characterized according to the color parameters: L = 66.06 a = 10.55; b = 1.93.

It can therefore be suggested that freeze-dried and micronized red potatoes of the Magenta Love variety are rich in polyphenols, health-promoting compounds, and essential nutrients, making them a suitable raw material for gluten-free snack production.

### 2.2. The Effect of Micronized and Freeze-Dried Red Potatoes on Polyphenol Content and Antioxidant Activity in Gluten-Free Snacks

Considering that the Folin–Ciocalteu reagent reacts not only with polyphenols but also with vitamin C, alkaloids, amino acids, proteins, organic acids, and polysaccharides [47,48], total polyphenol content was measured using two methods. The first method employed the Folin–Ciocalteu reagent [49], while the second avoided its use (Mazza et al. [50], modified by Oomah et al. [51]). It was observed that extrudates containing freeze-dried and micronized red potatoes (Magenta Love variety) at levels of 10–40% showed significantly higher total polyphenol content compared to the control. Using the Folin–Ciocalteu reagent, the total polyphenol content increased 4-fold with a 10% red potato addition and up to 10.5-fold with a 40% addition, relative to the control extrudate containing rice flour, maltodextrin, and corn meal (in a 1:1:1 ratio) (Table 2). When measured without the Folin–Ciocalteu reagent, the polyphenol content in the extrudates with freeze-dried and micronized red potatoes increased 7.6-fold to 23-fold compared to the control (Table 2). However, the polyphenol levels were lower when measured without the Folin–Ciocalteu reagent (Mazza et al. [50], modified by Oomah et al. [51]), supporting the claim that the reagent reacts with compounds other than polyphenols.

In the case of flavonoids, even a 10% addition of micronized and freeze-dried red potatoes resulted in a 5-fold increase in flavonoid content in the snacks, while a 40% addition led to a 28-fold increase compared to the control (Table 2). A significant increase was also noted for phenolic acids in snacks with this addition as compared to control. The control extrudate, composed of rice flour, maltodextrin, and corn meal, contained only trace amounts of phenolic acids (originating from corn meal). Adding as little as 10% freeze-dried and micronized red potatoes into snacks caused a 5-fold increase in phenolic acid content relative to the control. A similar trend was observed for flavonols, which were initially present in very low amounts in the control. A 10% addition of freeze-dried and micronized red potatoes resulted in a 2.42-fold increase in flavonol content in the extrudates as compared to control extrudates (Table 2). Comparing the content of polyphenols (both with and without the Folin–Ciocalteu reagent), flavonols, and flavonoids in extrudates containing freeze-dried and micronized red potatoes analyzed in this study with those from a previous study by Gumul et al. [40], which used extrudates containing freeze-dried red potatoes without micronization, a 30–40% increase in polyphenol content and a 20% increase in flavonol and flavonoid content was observed in the extrudates where red potatoes underwent micronization.

The polyphenol content in extrudates with red potatoes, measured both with and without the Folin–Ciocalteu reagent, was significantly higher than expected based on the level of freeze-dried and micronized red potato addition. The same trend applied to flavonoids, except for the 10% addition, which was less pronounced. In contrast, phenolic acid and flavonol content were lower than anticipated based on the level of addition. This discrepancy could be explained by potential decarboxylation of phenolic acids into 4-vinylguaiacol or by flavonols binding to other food components, making them less extractable from the samples (Table 2).

While many researchers argue that extrusion can lead to the loss of polyphenols and flavonoids due to their degradation or polymerization with other compounds, reducing their extractability [52,53], as well as the decarboxylation of phenolic acids, others suggest a different perspective. According to some studies [54,55], optimizing extrusion parameters, such as low moisture content and high temperature, can increase the release of these compounds from the cell walls of the extruded material. A similar effect is observed with micronization, where mechanical fragmentation using a ball mill enables the depolymerization and release of specific polyphenol fractions [30,31,32]. The type, quantity, and form of the additive (e.g., micronized material) are equally critical. Properly selected additives can enhance the levels of bioactive compounds in extrudates, even compensating for phenolic losses during the barothermal process [56,57,58,59]. Thus, subjecting freeze-dried red potatoes of the Magenta Love variety to micronization prior to extrusion further facilitated the release of polyphenols and flavonoids. However, in the case of phenolic acids, their partial release during micronization and subsequent exposure to harsh extrusion conditions likely led to decarboxylation into 4-vinylguaiacol. This explains their reduced presence in extrudates, although their levels remained significantly higher compared to the control extrudates (Table 2 and Table 3).

Analyzing the profile of phenolic compounds in extrudates containing micronized and freeze-dried red potatoes of the Magenta Love variety using UPLC-PDA-MS/MS, it can be noted that even the control sample—made of corn meal, rice flour, and maltodextrins—contains phenolic acids. These are partly endogenous acids derived from corn, such as caffeoylglycerol, p-coumarylquinic acid, 2-O-p-coumarylglycerol, di-p-coumarylspermidine, and feruloylquinic acid (Table 3). During extrusion (low moisture, high temperature), some of these acids are released from cell walls, explaining their presence in the control sample. Extrudates with micronized and freeze-dried red potatoes (Magenta Love) showed high levels of chlorogenic, cryptochlorogenic, and neochlorogenic acids. These compounds were introduced by the potato additive, and their amounts increased with higher levels of potato inclusion (Table 3). However, the increase in these phenolic acids in extrudates with red potatoes relative to the control was not proportional to the level of potato added. This suggests that red potatoes improve their functional properties during micronization. The process redistributes insoluble fiber into soluble fiber, reduces lignin content, and alters granule morphology. Consequently, micronization transforms raw material into a new powder with superior technological and functional properties. Micronization can release phenolic acids, enhancing their extractability [32]. When such material is further processed via extrusion to produce snacks, the combined effects of low moisture, high temperature, pressure, and friction may further disintegrate cell walls composed of fiber fractions. This could release additional phenolic acids. However, phenolic acids released during micronization may undergo decarboxylation during extrusion, forming 4-vinylguaiacol. This transformation leads to a reduced phenolic acid content in extrudates with micronized red potatoes. Nevertheless, the phenolic acid content in extrudates with freeze-dried and micronized red potatoes remains significantly higher than in the control (Table 3). Phenolic acids, such as neochlorogenic, cryptochlorogenic, and the dominant chlorogenic acid, are present in extrudates only after the addition of micronized and freeze-dried red potatoes. The lowest levels of these acids were found in extrudates with 10% red potato inclusion, while the highest levels were observed in those with 40% inclusion. The phenolic acid content in extrudates with 40% Magenta Love red potatoes was up to 3.5 times higher than in those with a 10% addition of red potatoes (Table 3).

The amount of remaining phenolic acids (caffeoylglycerol, p-coumarylquinic acid, 2-O-p-coumarylglycerol, di-p-coumarylspermidine, and feruloylquinic acid) in extrudates with red potatoes is influenced by the use of corn meal. Their levels decrease when corn meal, a component of the base mixture, is replaced with red potatoes (Table 3).

For anthocyanins, their content in extrudates was found to be proportional or even higher than the level of freeze-dried and micronized red potato inclusion. Derivatives of pelargonidin and peonidin were identified in extrudates containing red potatoes, as these compounds originate from the added potatoes. A threefold increase in these anthocyanins was observed in extrudates with 40% inclusion of micronized and freeze-dried Magenta Love red potatoes compared to those with 10% addition. The significant increase in anthocyanins in extrudates containing micronized and freeze-dried red potatoes, compared to control extrudates, can be attributed to the greater stability of anthocyanins derived from red potatoes compared to those from colorful fruits. Anthocyanins in red potatoes are typically acetylated with p-coumaric or ferulic acid, enhancing their stability during high-temperature processes and storage [60,61]. This property is particularly important, as many researchers argue that anthocyanins are inherently unstable due to factors such as pH, temperature, light, oxygen, and interactions with other components in food matrices. Anthocyanins found in red potatoes exhibit stability, which can be attributed to specific chemical mechanisms. These include the interaction between the acyl groups and the pyrylium ring of the flavylium cation, which diminishes the likelihood of water acting as a nucleophile on the hydrophilic anthocyanin molecules. This, in turn, reduces the formation of both the colorless pseudobase and the light yellow chalcone forms [62,63]. Polyphenolic compounds in potato tubers are associated with macromolecules such as polysaccharides or dietary fiber. These interactions involve hydrogen bonds or hydrophobic forces [64,65]. Some studies suggest these bonds might even be covalent [66]. Anthocyanins likely interact with other matrix components through ionic forces [67]. Most researchers agree that the binding capacity of phenolic compounds to starch and non-starch polysaccharides is influenced by their molecular weight [64,68,69]. In contrast, Jakobek [70] highlighted additional factors such as the stereochemistry of phenolic compounds, glycosylation degree, and the number of hydroxyl groups. It can be suggested that the micronization of freeze-dried red potatoes had less impact on releasing these compounds compared to extrusion (the combined parameters in this process: high temperature, high pressure, and shear forces). Extrusion facilitated their release, enhancing extraction and influencing the assay results. For phenolic acids, primarily bound via weak hydrogen bonds, micronization likely liberated these compounds. Subsequent extrusion likely caused decarboxylation of some phenolic acids into 4-vinyl derivatives, resulting in less proportional increases than expected from the red potato addition. A low increase in phenolic acid levels, after the added red potato into extrudates, was observed using both the Mazza et al. [50] method modified by Omaha [51] and chromatographic analysis (Table 2 and Table 3). However, the significant rise in anthocyanin content in extrudates with micronized freeze-dried red potatoes is a valuable finding. Anthocyanins possess exceptional health-promoting properties, including anti-inflammatory, antiviral, and antibacterial effects. They reduce risks of cancer, coronary disease, neurodegenerative disorders (e.g., Alzheimer’s and Parkinson’s diseases), diabetes, and cataracts. These compounds exhibit strong antioxidant activity, enhancing the health potential of products containing them [4,16,17].

**Figure 4:**
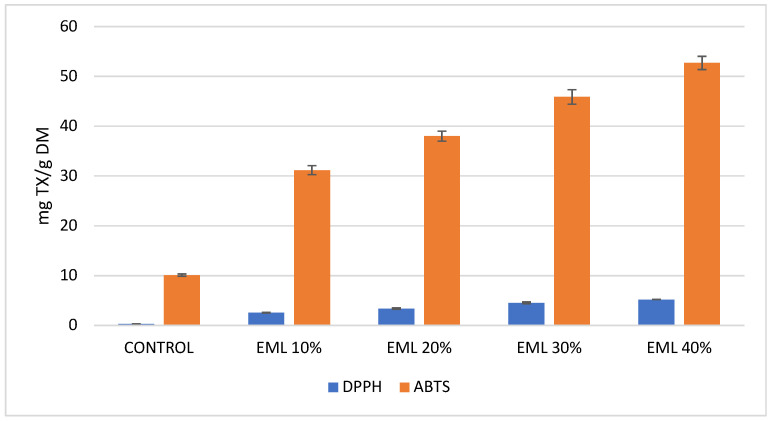
Antiradical activity of extrudates enriched with micronized and freeze-dried Magenta Love red potatoes.

Considering the antiradical activity measured against DPPH and ABTS radicals, it can be unequivocally stated that extrudates containing 10% to 40% of micronized and freeze-dried Magenta Love potatoes exhibited significantly higher activity compared to the control sample. Analysis of antiradical activity using DPPH revealed that the results were markedly lower than those obtained with ABTS (Figure 4). This discrepancy is likely due to the interference of other compounds, such as carotenoids, which absorb at the same wavelength in this assay [71,72]. Potatoes are an excellent source of carotenoids, which are lipophilic compounds. These include a wide group of substances such as lutein, zeaxanthin, neoxanthin, and beta-carotene [73]. As mentioned earlier, these compounds may cause inaccuracies in antioxidant activity measurements with DPPH. Therefore, the ABTS method was employed as an alternative. Even the smallest addition of 10% micronized, freeze-dried Magenta Love red potatoes to extrudates resulted in an eightfold increase in antiradical activity measured with the DPPH method. A 40% addition led to a 16-fold increase compared to the control. For the ABTS assay, the antiradical activity of extrudates with these potatoes increased three- to fivefold relative to the control (Figure 4).

According to various authors [9,15,74,75], the antioxidant activity of red- and purple-fleshed potatoes is primarily attributed to anthocyanins. However, Stushnoff et al. [76] note that chlorogenic acid and its two isomers, neochlorogenic and cryptochlorogenic acids, also play a significant role in the antioxidant activity of potatoes. Moreover, anthocyanins exhibit a synergistic effect with these phenolic acids. The antioxidant activity of anthocyanins is influenced by the degree of hydroxylation and methoxylation of their aromatic ring [15]. The high levels of chlorogenic, neochlorogenic, and cryptochlorogenic acids, combined with the substantial increase in anthocyanins (especially with 10–40% potato inclusion), ensured the strong antioxidant potential of these extrudates compared to the control. These research dependencies are confirmed by strong correlations between DPPH and the content of neochlorogenic acid (R^2^ = 0.989); DPPH and chlorogenic acid (R^2^ = 0.968); DPPH and cryptochlorogenic acid (R^2^ = 0.977); as well as DPPH and anthocyanins (R^2^ = 0.986).Similarly, strong correlations were calculated between ABTS and the content of neochlorogenic acid (R^2^ = 0.982); ABTS and chlorogenic acid (R^2^ = 0.959); ABTS and cryptochlorogenic acid (R^2^ = 0.972); and ABTS and anthocyanins (R^2^ = 0.980). Thus, micronizing red potatoes before adding them to extrudates significantly enhanced the antioxidant potential of the snacks (Figure 4) compared to the results reported by Gumul et al. [40].

### 2.3. The Influence of Micronized and Freeze-Dried Red Potatoes on the Phytosterol and Tocopherol Content of Gluten-Free Snacks

Tocopherols and tocotrienols are well-known bioactive compounds found in plant materials [25,77,78]. Phytosterols are also vital bioactive compounds in plants [25,26,79]. This study assessed the levels of both compound groups. Potatoes are rich in alpha-tocopherol, with levels reaching up to 1 mg per 100 g of dry matter [25,78]. Thus, it is unsurprising that alpha-tocopherol content in extrudates increased with the addition of red potatoes, starting at a 20% inclusion level (Table 4). In contrast, gamma-tocopherol levels remained constant in both control extrudates and those containing freeze-dried and micronized red potatoes (Table 4). This stability is attributed to the primary source of gamma-tocopherol being corn meal [80]. Red potatoes do not contain gamma-tocopherol, explaining the lack of variation. According to Shahidi et al. [81], extrusion processes reduce tocopherol levels due to chemical changes such as thermal degradation, depolymerization, and recombination under high temperature, pressure, and shear forces. Tiwari and Cummins [82] and Zielinski et al. [83] suggests that lower extrusion temperatures result in significant losses (up to 91%), while higher temperatures may reduce these losses and stabilize tocopherols. In this study, the higher extrusion temperature (160–180 °C) likely minimized tocopherol losses.

Significant amounts of beta-sitosterol were observed in extrudates containing red potatoes. Even a 10% addition of freeze-dried micronized red potatoes nearly doubled beta-sitosterol levels, while a 40% addition led to a tenfold increase compared to the control (Table 4). This is due to the naturally high beta-sitosterol content in red potatoes [25,26]. Incorporating these potatoes into extrudates significantly enhanced beta-sitosterol levels. A similar pattern was observed for stigmasterol, also derived from red potatoes [25,26]. However, a threefold increase in stigmasterol content was achieved only with 30% or 40% additions of freeze-dried micronized red potatoes into extrudates (Table 4).

### 2.4. The Impact of Micronized and Freeze-Dried Red Potatoes on the Chemical Composition of Gluten-Free Snacks and Color Parameters of Final Products

Extrudates containing freeze-dried micronized red potatoes showed a 3- to 14-fold increase in protein content compared to the control (Table 5), reflecting the high protein levels in red potatoes. Such a significant increase in protein content in gluten-free snacks containing micronized and freeze-dried red potatoes, compared to the control extrudate, can be attributed to the incorporation of red potatoes, which are a rich source of high-quality protein with high biological value [84]. It should be noted that during extrusion, protein degradation may occur, leading to losses largely due to the formation of Maillard reaction products and protein–lipid complexes [85,86]. Therefore, the inclusion of red potatoes in extruded products appears to have a highly beneficial effect. Moreover, no changes in fat content were observed in extrudates containing 10–20% of freeze-dried and micronized red potatoes. This may be due to the formation of protein–lipid and starch–lipid complexes [85]. An increase in fat content was only noted in extrudates with the addition of higher levels (30% and 40%) of freeze-dried and micronized red potatoes, resulting in an increase of 23% and 49%, respectively, in the fat content of these products. The ash content rose significantly, ranging from 4.5- to 22.5-fold higher than the control. The amount of insoluble dietary fiber in the extrudates ranged from 0.09 to 1.20 g/100 g DM and increased progressively with higher red potato content. Soluble fiber levels were 10 times higher in extrudates with a 10% addition of red potatoes and 26 times higher with a 40% addition, compared to the control. Total fiber content also increased sharply in extrudates (with an 11-fold rise at 10% addition and a 50-fold increase at 40% addition) compared to the control snacks (Table 5). It should be emphasized that during extrusion, insoluble dietary fiber is partially converted into its soluble fraction [86]. This explains the significant increase in the soluble dietary fiber content observed after the addition of freeze-dried and micronized red potatoes into the extrudates (Table 5). This represents a notable added value of such products, as the soluble fiber fraction exhibits hypocholesterolemic, hypoglycemic, and anticarcinogenic effects [44,45,46].

Total sugar and starch contents decreased with the inclusion of red potatoes into extrudates, as the control was composed primarily of starchy ingredients such as maltodextrin, rice flour, and corn meal. Replacing these with red potatoes reduced sugar and starch levels, enhancing the nutritional profile. The loss of starch may also be attributed to the formation of its hydrolysis products, namely, high- and low-molecular-weight dextrins, as well as the fragmentation of starch polymers caused by shear forces within the extruder [87]. Moreover, during extrusion, starch undergoes partial gelatinization and melting. These processes involve a series of endothermic transformations within the starch molecule, which make it more reactive and capable of interacting with other components such as proteins, lipids, and macro- and microelements. Such interactions lead to the formation of starch–component complexes, including resistant starch fractions, which may contribute to an increase in the total dietary fiber content of the extruded products. At the same time, they may partially explain the observed reduction in starch content [88,89].

The extrudates with red potatoes exhibited higher levels of total fiber (especially soluble fraction), protein, and minerals, significantly improving their dietary value (Table 5).

Corn-based extrudates, commonly known as snacks or ready-to-eat (RTE) products, are widely recognized for their high sugar and starch content, resulting in very low nutritional value. These snacks are unsuitable for individuals with diabetes and may increase the risk of metabolic disorders such as obesity, type 2 diabetes, and cardiovascular diseases. Dietary fiber (DF), often naturally associated with polyphenols, has been proposed as a potential starch replacer in cereal product reformulation to create healthier foods [90,91,92,93]. Developing a new formulation for these snacks—one that significantly enhances their nutritional value and incorporates the proven health benefits of micronized and freeze-dried Magenta Love red potatoes—is a valuable outcome of this research. This represents an additional advantage supporting the inclusion of freeze-dried and micronized red potatoes in snack products. According to the NOVA classification [94], snacks are typically categorized as ultra-processed foods, which are generally not favored by dietitians. The incorporation of micronized and freeze-dried red potatoes into snacks aims to enrich them with high levels of polyphenols, phytosterols, and dietary fiber (particularly its soluble fraction), significantly improving the bioaccessibility and bioavailability of these health-promoting compounds. Although snacks are not a key component of a gluten-free diet, they remain highly popular among consumers. Therefore, the development of new products of this type is particularly important.

The analyses showed significant differences in the color parameters of the extrudate samples, measured using the CIE Lab* system, depending on the proportion of plant material. The ∆E00 value, which indicates perceptibility of color changes based on a more accurate and perceptually uniform metric than ΔEab, showed systematic increases with higher plant material content, reflecting growing intensity of color change compared to the control sample. For all samples with red potato addition, ΔE00 values were significantly above 10, denoting noticeable and significant color differences compared to the control. The pure addition sample (ML as an addition was characterized by color parameters L (66.06), a (10.55), b (1.93)), which was not subjected to extrusion, had a darker color similar to the extrudate with a 10% addition (similar L* value), with a prominent red color and a warm tone (high a* value indicating red and low b* value suggesting a minimal presence of yellow) [95]. Samples with increasing amounts of micronized and freeze-dried Magenta Love red potatoes showed a decrease in brightness and an increase in the intensity of red and yellow hues. The highest color intensity was observed in the 40% sample, which was the darkest and had the warmest color. The ΔE00 value for the 40% sample (26.72) confirmed the most significant perceptual difference in color compared to the control, correlating with its darker and warmer visual appearance.

Extrusion significantly affected the color changes of the samples, linked to Maillard reactions and caramelization of sugars occurring during high-temperature, high-pressure processing [96]. The addition of freeze-dried, micronized red potatoes significantly influenced the chemical composition of the extrudates, indirectly modifying their color. Extrudates with red potatoes showed up to a 14-fold increase in protein content compared to the control. The increase in protein and fiber, especially its soluble fraction (up to 26 times higher with a 40% potato addition compared to the control), likely intensified Maillard reactions, contributing to the darkening of the samples (Table 5 and Table 6). Fat content in the extrudates increased only at higher levels of potato addition (30% and 40%), which could further influence color by intensifying thermal reactions [97]. A reduction in sugar and starch content, resulting from replacing these components with red potatoes, may have somewhat limited caramelization, explaining the less intense darkening compared to sugar-rich materials. Increased ash content (up to 22.5 times higher in extrudates with the highest proportion of red potatoes) indicates a higher mineral content, which can also affect color through interactions with other components during extrusion [98] (Table 5 and Table 6). In conclusion, increasing the plant material content in the extrudate samples led to a systematic increase in perceptual color differences (ΔE00) compared to the control sample. The greatest changes were observed in the samples with the highest addition, with the 40% sample exhibiting the most pronounced color shift.

## 3. Materials and Methods

### 3.1. Materials

Potatoes of varieties Magenta Love—ML (red potato), were cultivated at the Department of Environmental Protection and Organic Farming in Spišská Belá, Slovakia. The harvested potatoes underwent freeze-drying for 40 h using a Gamma 1-16 LSC lyophilizer (Martin Christ Gefriertrocknungsanlagen GmbH, Osterode am Harz, Germany) under conditions of 20 °C shelf temperature and 0.1 mbar pressure. Following the freeze-drying process, the potatoes were ground with a Grindomix GM200 laboratory grinder (Retsch GmbH & Co. KG., Haan, Germany) and micronized using a ball mill (Pulverisette 6, Fritsch GmbH, Idar-Oberstein, Germany) at 300 rpm for 15 min, ensuring the temperature stayed below 30 °C.

Granule diameter and particle size distribution in the freeze-dried and micronized red potato variety Magenta Love were analyzed using laser particle size analyzer Analysette 22 NeXT (Fritsch GmbH, Idar-Oberstein, Germany) instruments. A sample (0.1 g) was weighed and dispersed in deionized water using a vortex mixer (WF2, Janke and Kunkel GmbH, Staufen im Breisgau, Germany) (10 s). The measurement was performed according to the standard operating procedure. Particle size distribution of the freeze-dried and micronized red potato variety was D.50 = 82.65 μm.

The processed samples were then used for extrudate production, which was subjected to further analysis.

### 3.2. Extrudate (Gluten-Free Snack) Production

Extrudates containing 10%, 20%, 30%, and 40% freeze-dried and micronized red potatoes derived from the Magenta Love variety (EML 10%; EML 20%; EML 30%; EML 40%), along with control extrudates (CONTROL), were prepared according to Table 7. Rice starch, maltodextrin (DE = 16), and corn meal (1:1:1 ratio) (premix) were ground to the required thickness before extrusion and then mixed. The moisture level in ingredients of the premix used for the extrusion was equilibrated at 14%, with a particle size of 500–850 μm. This extrudate served as the control. The rice starch and maltodextrin and corn meal mixture was then replaced with freeze-dried and micronized red potatoes in amounts of 10%, 20%, 30%, and 40%, resulting in red potato snacks (EML 10%–EML 40%). Extrusion was carried out using a twin-screw extruder (Fudex, model 2FS60, Cavriago, Italy). The screw speed was set to approximately 100 rpm. The screw diameter was 60 mm, and the extrusion process temperatures in different zones were as follows: zone I 140 °C/zone II 160 °C/zone III 130 °C, (compression ratio = 1:2). A die with two nozzles, each with a 3 mm diameter, was used.

### 3.3. Methods

The following analyses were performed on each sample of freeze-dried and micronized red potatoes, as well as the obtained extrudates:

#### 3.3.1. Determination of Basic Nutrients

The content of basic nutrients in the analyzed products was determined using AOAC methods [99]. Protein (N × 6.25) was measured by the Kjeldahl method [99] using a Kjeltec 2200 extraction unit (Foss Analytical, Hillerød, Denmark). Total carbohydrate content was determined by the AOAC method no. 974.06. Fat content was measured by the Soxhlet method (AOAC method no. 953.38) using a Soxtec Avanti 2055 unit (Foss Analytical, Hillerød, Denmark). Ash content was determined by AOAC method no. 930.05. The content of non-starch polysaccharides, i.e., total, soluble, and insoluble dietary fiber, was determined using the AACCI 32-07 method [100], and starch content was measured according to the ICC [101]. All of the above measurements were performed in at least two replicates.

#### 3.3.2. Analysis of Antioxidants and Antiradical Activity

The antioxidant compounds and antiradical activity were assessed in ethanol extracts. A 0.6 g sample was dissolved in 30 cm^3^ of 80% ethanol, shaken in the dark for 120 min (using an electric shaker, type WB22, Memmert, Schwabach, Germany), and then centrifuged for 15 min at 4000 rpm (MPW-350 centrifuge, MPW MED. Instruments, Warsaw, Poland). The supernatant was separated and stored at −20 °C for subsequent analysis. Total polyphenol content (TPC) was determined using two spectrophotometric methods: (1) with the Folin–Ciocalteu reagent, following Singleton et al. [49], and (2) without the Folin–Ciocalteu reagent, as per Mazza et al. [50], with modifications by Oomah et al. [51]. The phenolic acid content was measured spectrophotometrically, based on Mazza et al. [50] with Oomah et al. [51] modifications. Flavonol content was also determined spectrophotometrically, following the method by Mazza et al. [50] with modifications by Oomah et al. [51]. Flavonoid content was evaluated using the method by El Hariri et al. [102]. Antiradical activity was determined using the synthetic ABTS radical, following the method by Re et al. [103]. A diluted sample of the ethanol extract was mixed with ABTS, shaken on a vortex mixer (WF2, Janke and Kunkel GmbH, Staufen im Breisgau, Germany), and the absorbance was measured using a spectrophotometer (Helios Gamma, 100–240 Thermo Spectronic, Runcorn, UK) at λ = 734 nm. A second reading was taken after 6 min at the same wavelength. The results were expressed as mg/g dry matter (d.m.) Trolox equivalent (TEAC), with R^2^ = 0.999. Antioxidant activity in ethanol extracts was measured using 2,2-diphenyl-1-picrylhydrazyl (DPPH), according to Brand-Williams et al. [104]. Extracts (1 cm^3^) were mixed with 4 cm^3^ of DPPH solution (0.012 g DPPH in 100 cm^3^ ethanol). Absorbance was measured using a spectrophotometer at λ = 517 nm. Trolox (6-hydroxy-2,5,7,8-tetramethylchromano-2-carboxylic acid) was used as a standard, with R^2^ = 0.9829. Results were expressed as mg/g DM Trolox equivalent (TEAC).

#### 3.3.3. Determination of Polyphenolic Compounds by UPLC-PDA-MS/MS

Extraction: One gram of the sample was extracted with 10 cm^3^ of a mixture containing HPLC-grade methanol (30 cm^3^/100 cm^3^), ascorbic acid (2.0 g/100 cm^3^), and acetic acid (1.0 cm^3^/100 cm^3^). The extraction was performed twice by incubating the sample with sonication (Sonic 6D, Polsonic, Warsaw, Poland) for 20 min, mixing periodically. The suspension was centrifuged at 19,000× *g* for 10 min, and the supernatant was filtered through a hydrophilic PTFE membrane (0.20 μm) and used for analysis.

Analysis: Phenolic compounds were analyzed using an Aquity ultra-performance liquid chromatograph (Waters Corporation, Milford, MA, USA), equipped with a binary solvent manager (BSM) (Waters Corporation, Milford, MA, USA) and sample manager (SM) (Waters Corporation, Milford, MA, USA), connected to a PDA and Q-TOF mass detector (Waters, Manchester, UK). The analysis was performed on a UPLC BEH C18 column (2.1 × 100 mm, 1.7 μm particles, Waters). The elution was carried out with an isocratic gradient using 2% formic acid in water (A) and acetonitrile (B), with a flow rate of 0.45 mL/min. Elution began with 99% A for one minute, followed by a linear gradient to 75% B after 12 min. The column temperature was 30 °C, and the injection volume was 5 μL.

Mass Spectrometry: The mass detector was operated with a capillary voltage of 2.5 kV and a cone voltage of 30 V. The ion source and desolvation temperatures were set at 130 °C and 350 °C, respectively. Nitrogen was used as the carrier gas at a flow rate of 300 L/h. The analyses were performed in full scan mode (100–1500 *m*/*z*) with a resolution of 5000 and a tolerance of 0.001 Da. Internal reference standards, leucine and enkephalin, were introduced via the lockspray reference channel. Chromatograms were analyzed using base peak intensity (BPI) calibrated to 12,400 cps (100%). Data were collected and analyzed with MassLynx v4.1 software (Waters). Anthocyanins were analyzed in positive ion mode, while other polyphenols were analyzed in negative ion mode. Identification was based on UV absorption spectra, mass-to-charge ratio (*m*/*z*), retention times, and fragmentation spectra, compared with the available literature data. Fragmentation spectra were obtained by collision-induced dissociation (CID) in tandem mode. Collision energy was adjusted individually for each compound. UV spectra were collected at λ = 320 for phenolic acids, λ = 360 for flavonols, λ = 280 for flavan-3-ols, and λ = 340 for flavonones.

#### 3.3.4. Tocopherols and Phytosterols in Food Determination Using Gas Chromatography

Tocopherols and phytosterols in food determination using gas chromatography was studied by Hussain et al. [105], Oracz et al. [106], and Zhang et al. [107]. Samples were prepared by weighing 0.2 g of the sample (±0.0001 g) into a 20 mL vial. To this, 4 cm^3^ of freshly prepared saponification reagent (3.9 cm^3^ of 2 M KOH in methanol and 0.5 mL of 10% ascorbic acid) was added. The vial was sealed, incubated at 85 °C for 40 min, and then cooled to room temperature. The contents were transferred to 30 cm^3^ centrifuge tubes, with 10 cm^3^ of hexane and 10 cm^3^ of saturated NaCl solution. The tubes were tightly sealed, shaken for 10 min (175 rpm), and then centrifuged at 6000 rpm for 10 min. The upper hexane layer was collected, transferred to 20 cm^3^ vials, and evaporated under nitrogen. After drying, 1 cm^3^ of hexane was added, and the mixture sonicated for 10 s. The samples were filtered through a syringe nylon filter (0.2–0.45 µm pore size, ProSource Scientific, Calgary, Canada). Analysis was conducted using Shimadzu GC 2010 Plus Gas Chromatograph with FID detector (Shimadzu, Corp., Kyoto, Japan).

#### 3.3.5. Color Analysis

The color of the sample was evaluated using an instrumental method based on the Commission Internationale de l’Éclairage (CIE) Lab* system (CIE, http://www.cie.co.at, accessed on 15 March 2025). Reflectance in the CIE system [108] was measured with a spectrophotometer (Konica Minolta CM-3500d, Tokyo, Japan) at a 10° observer angle and a 30 mm slit width. Samples were placed in 55 mm diameter Petri dishes. The analysis determined the following parameters: L* (luminance, where L* = 0 represents black and L* = 100 represents white), a* (negative values indicate green and positive values indicate red), and b* (negative values indicate blue, and positive values indicate yellow). Each sample was measured in four replicates.

Color differences (ΔE00) between samples were calculated using the CIEDE2000 formula, which accounts for perceptual non-uniformities in color differences by incorporating corrections for lightness (ΔL′), chroma (ΔC′), and hue (ΔH′), as well as their interactions. This approach provides a more accurate representation of visual color differences than the ΔEab*, especially for small color differences or saturated colors [108].

$$\Delta E_{00}=\sqrt{{\left(\frac{\Delta L^{\prime }}{k_{L}S_{L}}\right)}^{2}+{\left(\frac{\Delta C^{\prime }}{k_{C}S_{C}}\right)}^{2}+{\left(\frac{\Delta H^{\prime }}{k_{H}S_{H}}\right)}^{2}+R_{T}\left(\frac{\Delta C^{\prime }}{k_{C}S_{C}}\right)\left(\frac{\Delta H^{\prime }}{k_{H}S_{H}}\right)}$$

where

ΔL′: Lightness difference.
ΔC′: Chroma difference.
ΔH′: Hue difference.
S_L_, S_C_, S_H_: Scaling functions for lightness, chroma, and hue, respectively.
R_T_: A rotation term accounting for hue and chroma interaction.
k_L_, k_C_, k_H_: Parametric weights (usually set to 1).

#### 3.3.6. Statistical Analysis

Experimental data were analyzed using one-way ANOVA (Duncan’s test) at a significance level of 0.05 with Statistica v. 8.0 software (StatSoft, Tulsa, OK, USA). All measurements were performed in at least two replicates. Results are presented as means with standard deviation. Additionally, Pearson correlation coefficients were calculated using Microsoft Excel for Microsoft 365 (Version 2403), based on selected data series. 

## 4. Conclusions

Extrudates containing micronized and freeze-dried red potatoes of the Magenta Love variety demonstrated high nutritional and health-promoting properties. Their nutritional and dietary value was attributed to a high protein and ash content, alongside significantly reduced levels of sugars and starch compared to the control sample. The addition of freeze-dried and micronized red potatoes into extrudates notably increased total dietary fiber and its two fractions (especially soluble fraction with a 26-fold increase in comparison to control), enhancing the health benefits of the final product.

The inclusion of freeze-dried and micronized Magenta Love red potatoes led to a high concentration of anthocyanins. These compounds were released from the nutritional matrix during micronization and extrusion processes. The extrudates also contained tocopherols and substantial levels of β-sitosterol and stigmasterol compared to the control.

Micronization of freeze-dried red potatoes used in gluten-free extrudates significantly improved nutritional and especially health-promoting qualities. This improvement was primarily due to the marked increase in dietary fiber and anthocyanins, with additional contributions from phenolic acids and also a huge antioxidant capacity.

The study conclusively demonstrated that the 40% addition of freeze-dried and micronized red potatoes to gluten-free extrudates ensures the development of an innovative product with excellent pro-health benefits and strong antioxidant activity.

This outlines a potential research strategy focused on the combined application of extrusion/micronization to reuse food processing by-products, which aligns perfectly with zero-waste technologies and the principles of sustainable food production.

## Figures and Tables

**Table 1:** Content of phenolic acids, anthocyanins, tocopherols, and sterols of freeze-dried and micronized red potatoes, variety ML (mg/100 g DM).

Phenolic Acids	
Neochlorogenic acid	287.00 ± 2.03
Chlorogenic acid	892.02 ± 1.42
Cryptochlorogenic acid	195.07 ± 0.81
p-Coumaric acid glucoside	12.13 ± 1.07
Anthocyanins	
Pelargonidin-3-O-(rutoside)-5-O-glucoside	9.74 ± 0.00
Peonidin-3-O-(rutoside)-5-O-glucoside	0.49 ± 0.04
Pelargonidin-3-(caffeoyl) rutinoside-5-glucoside	10.34 ± 0.12
Pelargonidin-3-O-(p-coumaroyl rutinoside)-5-O-glucoside	187.77 ± 1.23
Tocopherols and Sterols	
Alpha-tocopherol	0.71 ± 0.08
Stigmasterol	1.13 ± 0.11
Sitosterol	1.82 ± 0.12

**Table 2:** Phenolic compounds in extrudates enriched with micronized and freeze-dried Magenta Love red potatoes (data presented as equivalents in mg/g DM for each compound).

Samples	TPC (with F-C) (mg Catechin/g DM)	TPC (Without F-C) (mg Catechin/g DM)	Phenolic Acids (mg Ferulic Acid/g DM)	Flavonols (mg Quercetin/g DM)	Flavonoids (mg Rutin/g DM)
CONTROL	0.89 ± 0.00 ^a^ *	0.37 ± 0.02 ^a^	0.05 ± 0.00 ^a^	0.07 ± 0.00 ^a^	0.17 ± 0.00 ^a^
EML 10%	3.44 ± 0.12 ^b^	2.82 ± 0.00 ^b^	0.25 ± 0.00 ^b^	0.17 ± 0.08 ^b^	0.88 ± 0.00 ^b^
EML 20%	6.02 ± 0.20 ^c^	5.66 ± 0.92 ^c^	0.76 ± 0.08 ^c^	0.35 ± 0.05 ^c^	2.39 ± 0.09 ^c^
EML 30%	7.86 ± 0.16 ^d^	6.06 ± 0.56 ^c^	0.97 ± 0.03 ^d^	0.41 ± 0.03 ^c^	3.61 ± 0.02 ^d^
EML 40%	9.45 ± 0.13 ^e^	8.57 ± 0.63 ^d^	1.27 ± 0.06 ^e^	0.68 ± 0.04 ^d^	4.83 ± 0.03 ^e^

* Presented data are mean values ± standard deviation. Values assigned the same letters in particular columns are not significant at 0.05 level of confidence.

**Table 3:** Profile of phenolic compounds in extrudates enriched with micronized and freeze-dried Magenta Love red potatoes [mg/100 g DM].

Category	Compound	CONTROL	EML 10%	EML 20%	EML 30%	EML 40%
Phenolic derivatives	Caffeoylglicerol	2.07 ± 0.07 ^c^ *	1.87 ± 0.15 ^c^	1.61 ± 0.17 ^c^	1.21 ± 0.00 ^b^	1.00 ± 0.12 ^a^
	p-Coumaryl quinic acid	0.74 ± 0.05 ^e^	0.59 ± 0.00 ^d^	0.42 ± 0.00 ^c^	0.37 ± 0.01 ^b^	0.27 ± 0.00 ^a^
	2-O-P-Coumaryl glycerol	1.13 ± 0.11 ^c^	1.00 ± 0.13 ^c^	0.87 ± 0.03 ^b^	0.62 ± 0.09 ^a^	0.53 ± 0.00 ^a^
	Di-Coumaryl spermidine	2.89 ± 0.13 ^c^	2.47 ± 0.17 ^b^	2.03 ± 0.11 ^a^	1.95 ± 0.11 ^a^	1.71 ± 0.07 ^a^
	Feruloyl quinic acid	0.59 ± 0.00 ^e^	0.50 ± 0.00 ^d^	0.42 ± 0.02 ^c^	0.35 ± 0.00 ^b^	0.29 ± 0.01 ^a^
Anthocyanins and their glycosides	Pelargonidin-3-O-(rutoside)-5-O-glucoside	-	1.89 ± 0.19 ^a^	2.97 ± 0.11 ^b^	3.71 ± 0.13 ^c^	4.75 ± 0.00 ^d^
	Peonidin-3-O-(rutoside)-5-O-glucoside	-	0.07 ± 0.00 ^a^	0.11 ± 0.00 ^b^	0.19 ± 0.04 ^c^	0.37 ± 0.00 ^d^
	pelargonidin-3-(caffeoyl) rutinoside-5-glucoside	-	1.48 ± 0.20 ^a^	2.54 ± 0.14 ^b^	3.99 ± 0.00 ^c^	4.82 ± 0.13 ^d^
	Pelargonidin-3-O-(p-coumaroyl rutinoside)-5-O-glucoside	-	25.12 ± 1.02 ^a^	43.23 ± 0.93 ^b^	64.47 ± 1.02 ^c^	83.52 ± 1.49 ^d^
Phenolic acids	Neochlorogenic acid	-	30.08 ± 1.13 ^a^	57.02 ± 0.87 ^b^	79.13 ± 0.00 ^c^	94.08 ± 0.00 ^d^
	Chlorogenic acid	-	82.19 ± 1.09 ^a^	173.08 ± 0.43 ^b^	258.20 ± 1.15 ^c^	352.20 ± 1.02 ^d^
	Cryptochlorogenic acid	-	21.15 ± 0.75 ^a^	43.90 ± 0.49 ^b^	54.17 ± 1.20 ^c^	74.69 ± 2.41 ^d^
	p-Coumaric acid glucoside	-	1.17 ± 0.23 ^a^	2.41 ± 0.00 ^b^	3.27 ± 0.00 ^c^	4.31 ± 0.57 ^d^

* Presented data are mean values ± standard deviation. Values assigned the same letters in particular rows are not significant at 0.05 level of confidence.

**Table 4:** Sterols and Tocopherols in extrudates enriched with micronized and freeze-dried Magenta Love red potatoes.

Samples	Stigmasterol	β-Sitosterol	α-Tocopherol	γ-Tocopherol
CONTROL	0.33 ± 0.01 ^b^ *	0.84 ± 0.02 ^a^	0.00 ± 0.00	0.20 ± 0.01 ^a^
EML 10%	0.23 ± 0.01 ^a^	1.54 ± 0.14 ^b^	0.00 ± 0.00	0.21 ± 0.00 ^a^
EML 20%	0.37 ± 0.03 ^b^	4.49 ± 0.01 ^c^	0.16 ± 0.02	0.23 ± 0.01 ^ab^
EML 30%	1.01 ± 0.03 ^c^	8.51 ± 0.77 ^d^	0.26 ± 0.01	0.33 ± 0.19 ^ab^
EML 40%	1.00 ± 0.00 ^c^	9.01 ± 0.01 ^d^	0.31 ± 0.01	0.40 ± 0.01 ^ab^

* Presented data are mean values ± standard deviation. Values assigned the same letters in particular columns are not significant at 0.05 level of confidence.

**Table 5:** Chemical composition (g/100 g DM) of extrudates enriched with micronized and freeze-dried Magenta Love red potatoes.

Samples	Protein	Fat	Ash	Dietary Fiber—Insoluble Fraction	Dietary Fiber—Soluble Fraction	Dietary Fiber—Total	Total Sugars	Starch
CONTROL	0.28 ± 0.02 ^a^ *	0.34 ± 0.05 ^a^	0.12 ± 0.00 ^a^	0.00 ± 0.00	0.04 ± 0.01 ^a^	0.04 ± 0.01 ^a^	10.43 ± 0.01 ^e^	89.26 ± 0.05 ^d^
EML 10%	1.04 ± 0.01 ^b^	0.71 ± 0.33 ^a^	0.54 ± 0.00 ^b^	0.09 ± 0.01 ^a^	0.48 ± 0.02 ^b^	0.57 ± 0.00 ^b^	10.14 ± 0.01 ^d^	83.63 ± 0.05 ^c^
EML 20%	1.84 ± 0.01 ^c^	1.12 ± 0.72 ^a^	1.07 ± 0.01 ^c^	0.41 ± 0.01 ^b^	0.61 ± 0.02 ^c^	1.02 ± 0.03 ^c^	9.30 ± 0.01 ^c^	76.63 ± 0.35 ^b^
EML 30%	2.90 ± 0.09 ^d^	1.69 ± 1.21 ^b^	1.98 ± 0.03 ^d^	0.76 ± 0.03 ^c^	1.15 ± 0.02 ^d^	1.90 ± 0.01 ^d^	8.54 ± 0.02 ^b^	74.94 ± 0.05 ^a^
EML 40%	3.93 ± 0.04 ^e^	2.25 ± 1.68 ^c^	2.69 ± 0.01 ^e^	1.19 ± 0.04 ^d^	1.31 ± 0.03 ^e^	2.50 ± 0.00 ^e^	5.67 ± 0.01 ^a^	74.66 ± 0.06 ^a^

* Presented data are mean values ± standard deviation. Values assigned the same letters in particular columns are not significant at 0.05 level of confidence.

**Table 6:** Color parameters of snacks with micronized and freeze-dried red potatoes.

Samples	L* (D65)	a* (D65)	b* (D65)	ΔE_00_
CONTROL	79.40 ± 0.03 ^c^ *	1.92 ± 0.02 ^a^	13.20 ± 0.15 ^b^	-
EML 10%	68.36 ± 0.03 ^a^	7.21 ± 0.01 ^c^	25.11 ± 0.05 ^d^	17.64 ± 0.00 ^b^
EML 20%	61.83 ± 0.20 ^a^	9.07 ± 0.10 ^d^	26.42 ± 0.17 ^e^	23.80 ± 0.00 ^c^
EML 30%	59.45 ± 0.04 ^a^	9.48 ± 0.03 ^d^	25.63 ± 0.08 ^de^	25.42 ± 0.00 ^d^
EML 40%	59.06 ± 0.07 ^a^	10.25 ± 0.03 ^e^	27.01 ± 0.06 ^f^	26.72 ± 0.00 ^e^

* Presented data are mean values ±standard deviation. Values assigned the same letters in particular columns are not significant at 0.05 level of confidence.

**Table 7:** Recipe for extrudate production with varying levels of micronized freeze-dried red potatoes.

Sample Name	Corn Meal/Rice Starch/Maltodextrin (1:1:1) (g)	Freeze-Dried/Micronized Red Potato (g)
CONTROL	5000	0
EML 10%	4500	500
EML 20%	4000	1000
EML 30%	3500	1500
EML 40%	3000	2000

## Data Availability

Data is contained within the article.

## Abbreviations

The following abbreviations are used in this manuscript:

EML 10%	Extrudates containing 10% micronized and freeze-dried red potatoes derived from Magenta Love variety
EML 20%	Extrudates containing 20% micronized and freeze-dried red potatoes derived from Magenta Love variety
EML 30%	Extrudates containing 30% micronized and freeze-dried red potatoes derived from Magenta Love variety
EML 40%	Extrudates containing 40% micronized and freeze-dried red potatoes derived from Magenta Love variety
